# Identification of potential resistance mechanisms and therapeutic targets for the relapse of BCMA CAR-T therapy in relapsed/refractory multiple myeloma through single-cell sequencing

**DOI:** 10.1186/s40164-023-00402-5

**Published:** 2023-05-08

**Authors:** Wei Li, Binglei Zhang, Weijie Cao, Wenli Zhang, Tiandong Li, Lina Liu, LinPing Xu, Fengcai Gao, Yanmei Wang, Fang Wang, Haizhou Xing, Zhongxing Jiang, Jianxiang Shi, Zhilei Bian, Yongping Song

**Affiliations:** 1 Department of Hematology, The First Affiliated Hospital of Zhengzhou University, Zhengzhou, 450052 Henan China; 2 Department of Hematology, Henan Provincial Hematology Hospital, Zhengzhou, 450000 Henan China; 3 BGI College & Henan Institute of Medical and Pharmaceutical Sciences in Academy of Medical Science, Zhengzhou University, Zhengzhou, 450052 Henan China; 4 Department of Hematology, The Affiliated Cancer Hospital of Zhengzhou University, Zhengzhou, 450008 Henan China; 5 College of Public Health, Zhengzhou University, Zhengzhou, 450000 Henan China; 6 Department of Research and Foreign Affairs, The Affiliated Cancer Hospital of Zhengzhou University & Henan Cancer Hospital, Zhengzhou, 450008 China; 7 Department of Hematology, Zhengzhou People’s Hospital, Zhengzhou, 450003 Henan China

**Keywords:** BCMA CAR-T, Multiple myeloma, Resistant mechanisms, ScRNA-seq, T cell exhaustion, Monocyte/macrophages

## Abstract

**Background:**

BCMA CAR-T is highly effective for relapsed/refractory multiple myeloma(R/R-MM) and significantly improves the survival of patients. However, the short remission time and high relapse rate of MM patients treated with BCMA CAR-T remain bottlenecks that limit long-term survival. The immune microenvironment of the bone marrow (BM) in R/R-MM may be responsible for this. The present study aims to present an in-depth analysis of resistant mechanisms and to explore potential novel therapeutic targets for relapse of BCMA CAR-T treatment via single-cell RNA sequencing (scRNA-seq) of BM plasma cells and immune cells.

**Methods:**

This study used 10X Genomic scRNA-seq to identify cell populations in R/R-MM CD45^+^ BM cells before BCMA CAR-T treatment and relapse after BCMA CAR-T treatment. Cell Ranger pipeline and CellChat were used to perform detailed analysis.

**Results:**

We compared the heterogeneity of CD45^+^ BM cells before BCMA CAR-T treatment and relapse after BCMA CAR-T treatment. We found that the proportion of monocytes/macrophages increased, while the percentage of T cells decreased at relapse after BCMA CAR-T treatment. We then reclustered and analyzed the alterations in plasma cells, T cells, NK cells, DCs, neutrophils, and monocytes/macrophages in the BM microenvironment before BCMA CAR-T treatment and relapse after BCMA CAR-T treatment. We show here that the percentage of BCMA positive plasma cells increased at relapse after BCMA CAR-T cell therapy. Other targets such as CD38, CD24, SLAMF7, CD138, and GPRC5D were also found to be expressed in plasma cells of the R/R-MM patient at relapse after BCMA CAR-T cell therapy. Furthermore, exhausted T cells, TIGIT^+^NK cells, interferon-responsive DCs, and interferon-responsive neutrophils, increased in the R/R-MM patient at relapse after BCMA CAR-T cell treatment. Significantly, the proportion of IL1β^hi^ Mφ, S100A9^hi^ Mφ, interferon-responsive Mφ, CD16^hi^ Mφ, MARCO ^hi^ Mφ, and S100A11^hi^ Mφ significantly increased in the R/R-MM patient at relapse after BCMA CAR-T cell therapy. Cell–cell communication analysis indicated that monocytes/macrophages, especially the MIF and APRIL signaling pathway are key players in R/R-MM patient at relapse after BCMA CAR-T cell therapy.

**Conclusion:**

Taken together, our data extend the understanding of intrinsic and extrinsic relapse of BCMA CAR-T treatment in R/R-MM patient and the potential mechanisms involved in the alterations of antigens and the induced immunosuppressive microenvironment, which may provide a basis for the optimization of BCMA CAR-T strategies. Further studies should be performed to confirm these findings.

**Supplementary Information:**

The online version contains supplementary material available at 10.1186/s40164-023-00402-5.

## Introduction

Multiple myeloma (MM) is a common hematological malignancy that mainly occurs in the elderly and accounts for approximately 10% of hematological malignancies. It is the second most common malignancy of the hematologic system [1]. In recent years, next-generation drugs such as immunomodulators, proteasome inhibitors, and monoclonal antibodies have significantly improved the prognosis of MM patients, with the median survival has extended to 3–6 years [2]. However, MM remains an incurable malignant hematologic neoplasm. With each relapse, malignant plasma cells undergo clonal evolution and acquire new mutations that confer the high-risk characteristics of MM and its resistance to standard therapy [3]. Therefore, new measures that can induce sustained remission in MM are urgently needed.

In recent years, CAR-T cell immunotherapy targeting CD19 has gained momentum in the treatment of relapsed/refractory acute lymphoblastic leukemia and lymphoma [4–8]. Subsequently, CAR-T cells targeting BCMA have also made great progress in the treatment of relapsed/refractory multiple myeloma (R/R-MM) [9–14]. Recent domestic and international studies have reported significant efficacy of BCMA CAR-T in R/R-MM, with some reports of objective response rate (ORR) reaching 100% [9–14]. Although encouraging progress has been made in research in the field of MM with BCMA CAR-T cells treatment, the short remission time and high relapse rate of patients treated with BCMA CAR-T cells remain bottlenecks that limit long-term patient survival [15, 16]. Therefore, there is an urgent need to analyze the mechanism of BCMA CAR-T cell failure in R/R-MM patients precisely.

The most important technical advantage of single-cell sequencing (ScRNAseq) technology is that it can address the problem of cell population heterogeneity, and the heterogeneity of multiple tumor microenvironments has now been resolved with the use of this technology. With the use of ScRNA-seq, *Mehmet Kemal Samur *et al. have reported that a clone with biallelic loss of BCMA is a resistance mechanism to CAR-T cell therapy in patients with MM[17]. Another study has indicated that an underlying mechanism of CAR-T immunotherapy failure is the selection of a clone with homozygous deletion of TNFRSF17 (BCMA) [18]. However, to date, there is no clear understanding of the single immune cell alterations in the bone marrow (BM) microenvironment of R/R-MM patients who relapse after BCMA CAR-T cell therapy.

In this study, we present an in-depth analysis of CD45^+^ BM cells from the patients enrolled in the clinical study to evaluate BCMA CAR-T in patients with R/R-MM (ClinicalTrials.gov Identifier: NCT03661554). We show that positive relapse and negative relapse occur simultaneously with BCMA CAR T-cell therapy in the patient with an initial response who relapsed after 22 months. Furthermore, immune cells including T cells, NK cells, DCs, neutrophils, and monocytes/macrophages, present tumor-promoting phenotypes. Cell–cell communication analysis indicated that monocytes/macrophages, especially the MIF and APRIL signaling pathway are key players in the R/R-MM patient at relapse after BCMA CAR-T cell therapy. Our results reveal the underlying mechanism of R/R-MM relapse after BCMA CAR-T cell therapy, which may provide a theoretical basis for the exploration of new BCMA CAR-T cell therapeutic strategies.

## Materials and methods

### Patient samples

Our group launched a single-arm, single-center clinical trial on BCMA CAR-T cells in the treatment of R/R-MM on April 11, 2018. The construction of anti-BCMA CAR, lentivirus production and production of CAR-T cells were described by our previous study [11]. Thirty-four patients with R/R-MM were treated with BCMA CAR-T cell therapy and the preliminary results have been reported in the journal of *leukemia* (ClinicalTrials.gov Identifier: NCT03661554) [11]. The ORR is 88.2% and the median progression-free survival time (PFS) is 12.1 months [11]. To analyze the mechanism of relapse after BCMA CAR-T cell therapy, one patient at baseline (R/R-MM) and one patient at progression (first relapse after BCMA CAR-T therapy) were enrolled in the study. The patient relapse after BCMA CAR-T cell therapy is from one of the 34 patients, who was enrolled in this clinical trial on December 13, 2019, and a relapse was detected on October 27, 2021(namely as progression in this study). And the other patient is from the newly enrolled, who met the criteria for enrollment in the clinical trial on December 3, 2021, after screening (namely as baseline in this study). The detailed characteristics of these two patients including clinicopathological features, bone marrow plasma cell ratio, staging and so on were comparable and similar (Additional file 1: Table S1). Five milliliters (mL) of bone marrow were then extracted from the two patients, and the CD45^+^ cells were enriched with human CD45 microbeads, according to the manufacturer’s instructions (Miltenyi Biotec, Cat#:130-045-801). The CD45^+^ cells were subsequently sent for 10 × genomics ScRNA-seq.

### Single-cell RNA library preparation and sequencing

Fresh CD45^+^ cells enriched from two patients’ BM were resuspended in PBS with 0.04% bovine serum albumin to a final concentration of 700–1200 cells per μl. The Chromium Controller (10× Genomics) was used to generate single cell partitions and molecular barcoding. The Chromium Single Cell 3′ Reagent Kit v3.1 (10× Genomics) was used to generate single cell sequencing library. Eight thousand cells per sample were targeted in this study. All other steps were implemented according to the manufacturer’s protocols. Generated scRNA-seq libraries were run on the Illumina Novaseq (Zebrafish [Beijing] Technology Corporation, Beijing, China) for PE150 sequencing.

### ScRNA-seq data processing

Sample demultiplexing, barcode processing, alignment to the human genome (GRCh38) and raw gene expression counting were performed using the Cell Ranger (version 6.0.1) pipeline. Raw single cell expression matrices were further analyzed by R software (version 4.1.0) with the Seurat package (version 4.2.0). Ambient RNA was removed by using the soupX package (version 1.6.1). Low-quality cells were removed if they met any of the following criteria: (1) > 20% UMIs derived from the mitochondrial genome; (2) < 300 genes. Doublets were identified by the R package DoubletFinder (version 2.0.3) with default settings. After the removal of low-quality cells and doublets, 10,595 cells and 21,371 genes were kept for further analysis. SCTransform function from the Seurat package was used to normalize gene expression. ScRNA-seq data matrices from two samples were integrated by using FindIntegrationAnchors and IntegrateData functions to remove the batch effect across samples. The data were used in the subsequent nonlinear dimensional reduction with the RunUMAP function and cluster analysis by the FindNeighbors and FindClusters functions. All details regarding the Seurat analyses performed in this work can be found in the website tutorial. (https://satijalab.org/seurat/articles/pbmc3k_tutorial.html).

### Cell type annotation and cluster marker identification

Clusters were identified using Seurat k-nearest neighbors clustering with resolution 0.3. Cell types were assigned by examining the expression of marker genes and the top differentially expressed genes in each cluster. Cells were visualized by Uniform Manifold Approximation and Projection (UMAP) plot. The FindAllMarkers function from Seurat were used to identify the signature genes of each cell cluster. Major cell types were further sub-clustered to further revealing the mechanism of BCMA CAR-T treatment resistance in MM. In addition, the percentages of different cell types or cell subtypes were calculated accordingly.

### Differentially expressed genes (DEGs) identification and functional enrichment

DEGs were identified by using the FindAllMarkers function from Seurat package with the following parameters: ‘only.pos = T’, ‘min.pct = 0.1’, ‘logfc.threshold = 0.25’. Enrichment analysis of DEGs were conducted using the clusterProfiler package.

### Cell–cell communication analysis

Cell–cell communication tool, CellChat was used in the present study to identify the major signals for each cell groups and outgoing, incoming and global communication patterns as previous described [19]. The key signals and latent communication patterns among all signaling pathways were identified to each cell groups before and after failure of BCMA CAR-T cell therapy. The major signaling sources, targets, essential mediators and key influencers, as well as other high-order information in intercellular communications were also analyzed before and after failure of BCMA CAR-T cell therapy.

### Statistical analysis

R version 4.1.0. was used for all statistical analyses. A *P*-value < 0.05 was considered to be statistically different.

## Results

### Single-cell sequencing of the BM CD45^+^ cells in R/R-MM patient upon BCMA CAR-T cell therapy

**Fig. 1:**
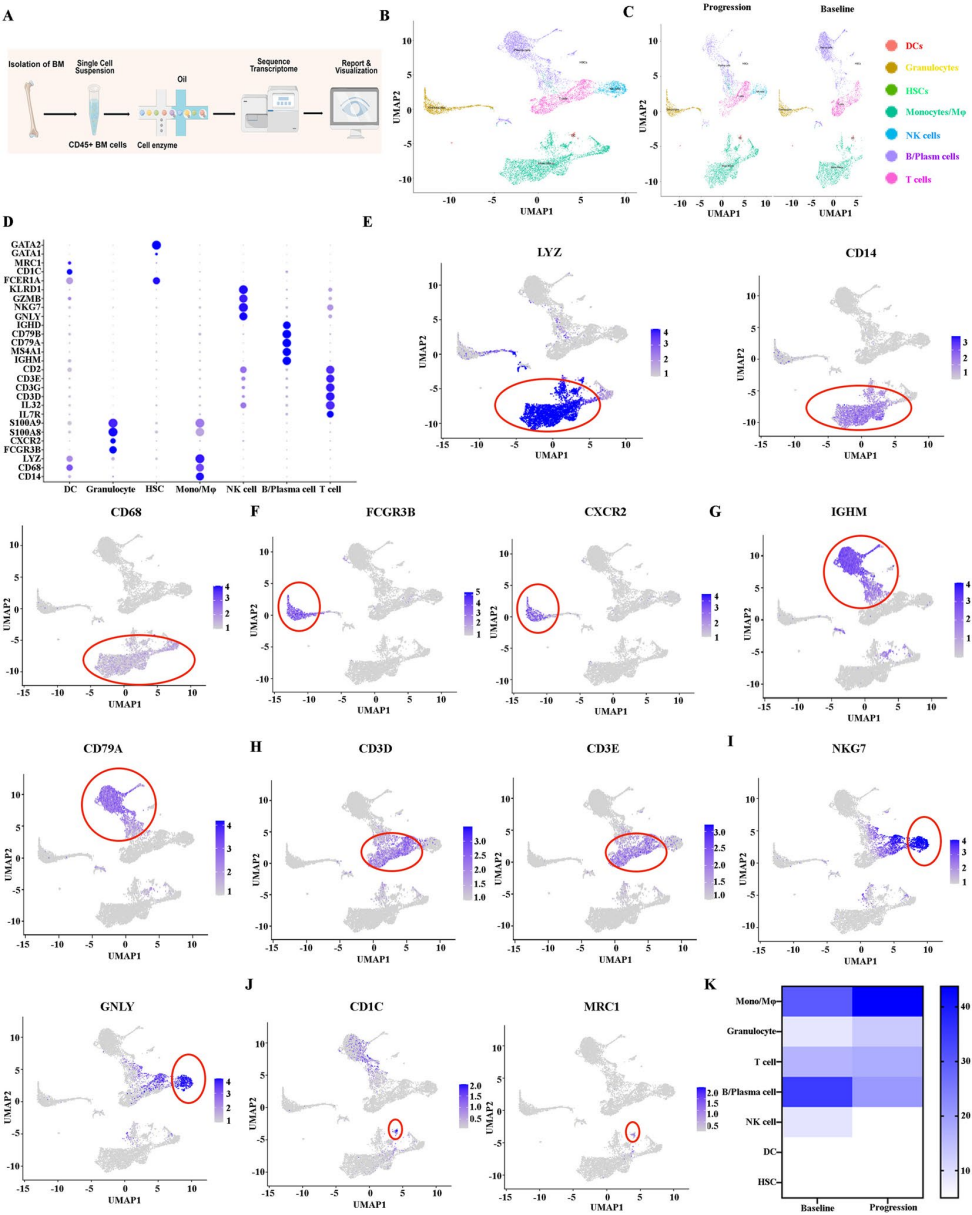
Single-cell sequencing reveals heterogeneous changes in the BM microenvironment of CD45^+^ cells in R/R-MM patients before and relapse after BCMA CAR-T cell therapy. **A** Single cell sequencing and analysis fluency chart. **B** The UMAP projection of CD45^+^ BM cells of the patients at baseline and progression, including T cells, B/plasm cells, NK cells, monocytes/macrophages, DCs, neutrophils, and HSCs.** C** The UMAP plots of the patients at baseline and progression. **D** Dot plot of differentially expressed genes in each cluster.** E** The UMAP plot of CD14, CD68, and LYZ expression of monocytes/macrophages. **F** The UMAP plot of FCGR3B and CXCR2 expression of neutrophils. **G** The UMAP plot of IGHM and CD79A expression of B/plasma cells. **H** The UMAP plot of CD3D and CD3E expression of T cells. **I** The UMAP plot of NKG7 and GZLY expression of NK cells. **J** The UMAP plot of CD1C and MRC1 expression of DC cells. **K** All the cell type proportions of R/R-MM patients at baseline and progression

To investigate the cellular diversity and molecular signatures before (baseline) and relapse after BCMA CAR-T cell therapy (progression) in R/R-MM patients, we generated scRNA-seq profiles from the patients at baseline and progression using 10× Genomics sequencing. We sequenced CD45^+^ BM cells obtained from the patients at baseline and progression. Figure 1A illustrates the flow diagram of single cell sequencing and analysis. After rigorous filtering, 5757 and 4838 CD45^+^cells at baseline and progression, respectively, were retained for further analysis. After normalization of gene expression, we performed dimensionality reduction and clustering using Uniform Manifold Approximation and Projection (UMAP; Fig. 1B). These cells from baseline and progression can be divided into seven different major cell types (Fig. 1C) with the use of well-known markers: T cells ( marked with CD3D, CD3E, CD3G), B cells/plasma cells (marked with CD79A, CD79B, IGHM, IGHD, MS4A1), NK cells (marked with GNLY, NKG7, GZMB, KLRD1), monocytes/macrophages (marked with CD14, CD68, LYZ), neutrophils (marked with S100A8, S100A9, CXCR2, FCGR3B), DCs (marked with CD1C,MRC1,FCER1A), and HSCs (marked with GATA2, GATA1; Fig. 1D–J). We then calculated the changes in the proportion of each cell type between baseline and progression. We found that the proportion of neutrophils (499 cells VS 625 cells; 8.67% VS 12.92%), monocytes/macrophages (1736 VS 2117 cells; 30.15% VS 43.76%) and HSCs (4 VS 14 cells, 0.07% VS 0.29%) increased, while T cells (939 VS 851 cells; 16.31% VS 17.59%), B cells/plasma cells (2,001 VS 990 cells; 34.76% VS 20.46%) and NK cells (522 VS 185cells; 9.07% VS 3.82%) decreased at relapse after BCMA CAR-T cell treatment (Fig. 1K). The differentially expressed genes (DEGs) and marker genes, as shown in the heatmap, confirmed the accuracy of cell identity (Additional file 8: Fig. S1).

### BCMA positive relapse and negative relapse occur concurrently at relapse after BCMA CAR-T cell therapy in R/R-MM

**Fig. 2:**
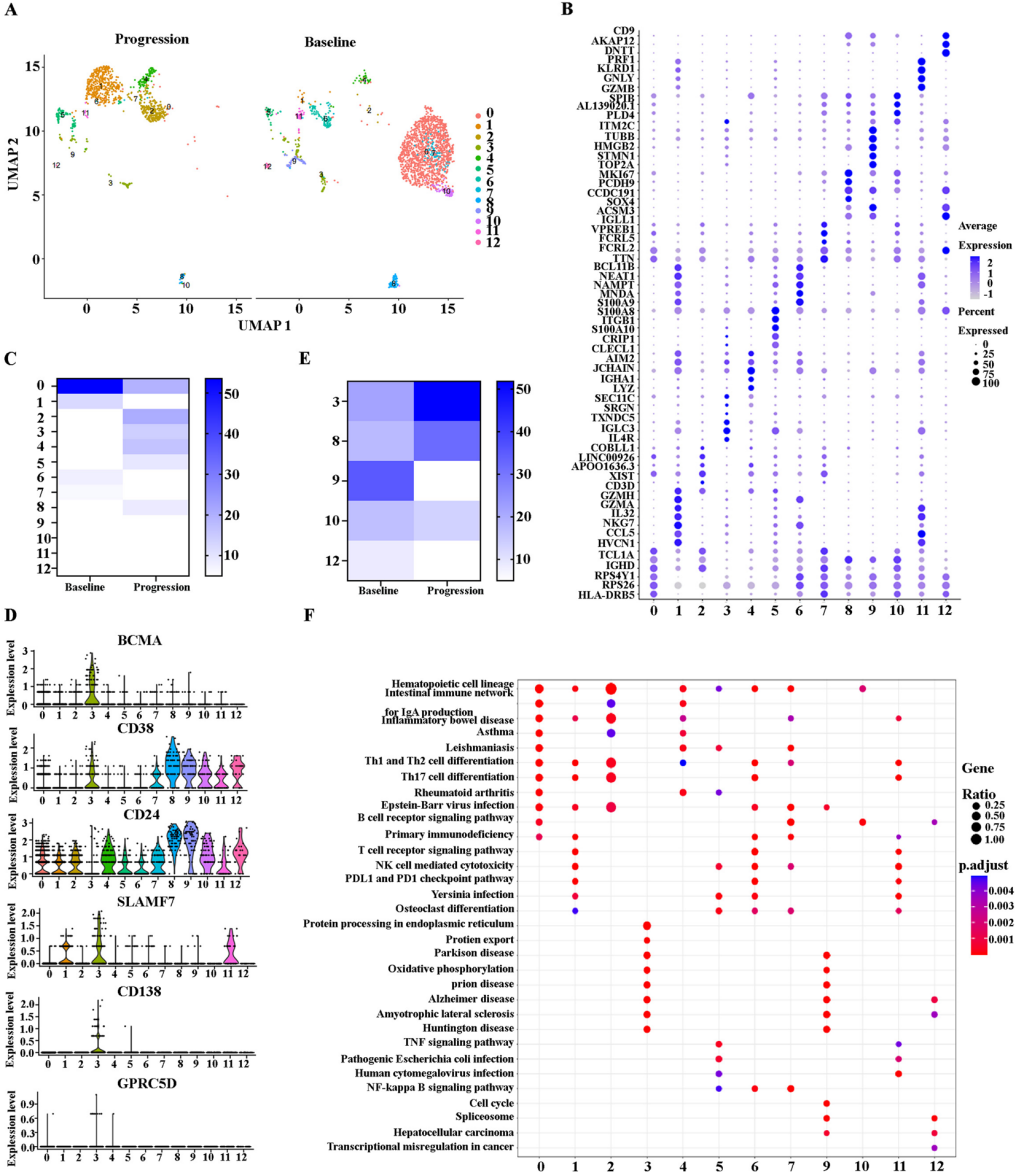
BCMA positive and negative relapses occur concurrently in at relapse after BCMA CAR-T cell therapy in R/R-MM. **A** The UMAP plots of B/plasma cells at baseline and progression. **B** Dot plot of differentially expressed genes in each cluster. **C** The proportions of B/plasma cells of all the clusters at baseline and progression.** D** The violin diagram of BCMA, CD38, CD24, SLAMF7, CD138, and GPRC5D expression in each B/plasma cell cluster.** E** The proportion of plasma cell cluster at baseline and progression. **F** The enrichment of KEGG pathway in each cluster of B/plasma cells

Previous studies have identified both negative and positive relapse mechanisms after CAR-T cell therapy [3, 17, 18, 20]. To clarify the mechanism of relapse after BCMA CAR-T cell treatment, we extracted B/plasma cells at baseline and progression for subpopulation reanalysis. We identified 13 main subclusters following the reclustering of B/plasma cells (Fig. 2A). A FeaturePlot of the top six marker genes of each cluster is shown in Fig. 2B**.** All the marker genes of these 13 clusters are demonstrated in Additional file 2: Table S2. The percentages of each cluster of the B cells/plasmas are shown in Fig. 2C. Of the 13 clusters, 3, 8, 9, 10, and 12 are malignant plasma cells. Cluster 3 expressed SDC1(CD138), MKI67, TNFRSF17(BCMA), and SLAMF7. Cluster 8 expressed CD38, CD24, and GAS7. Cluster 9 expressed CDK1, MKI67, TOP2A, and CD38. Cluster 10 expressed IGHM, CD38, and CD24. Cluster 12 expressed CD38 and CD24 (Fig. 2D). We then calculated the malignant plasma cell numbers of these five clusters and cell numbers at baseline, and the progression in clusters 3, 8, 9, 10, and 12 were 56 versus 141, 45 versus 87, 91 versus 7, 43 versus 36, and 22 versus 1, respectively. The percentages of clusters 3, 8, 9, 10, and 12 at baseline and progression among the plasma cells were 21.8% versus 51.8%, 17.5% versus 32%, 35.4% versus 2.6%, 16.7% versus 13.2%, and 8.6% versus 0.4%, respectively (Fig. 2E). A Kyoto Encyclopedia of Genes and Genomes (KEGG) analysis of Cluster 3 included protein processing in endoplasmic reticulum, protein export, and oxidative phosphorylation; the KEGG analysis of Cluster 9 included cell cycle and spliceosome; the KEGG analysis of Cluster 10 included the B cell receptor signaling pathway; and the KEGG analysis of Cluster 12 included transcriptional misregulation in cancer **(**Fig. 2F**)**. Taken together, our results indicate that BCMA positive and negative relapses occur concurrently at relapse after BCMA CAR-T cell therapy in R/R-MM.

### Significant increase in exhausted T cells at relapse after BCMA CAR-T cell therapy in R/R-MM

**Fig. 3:**
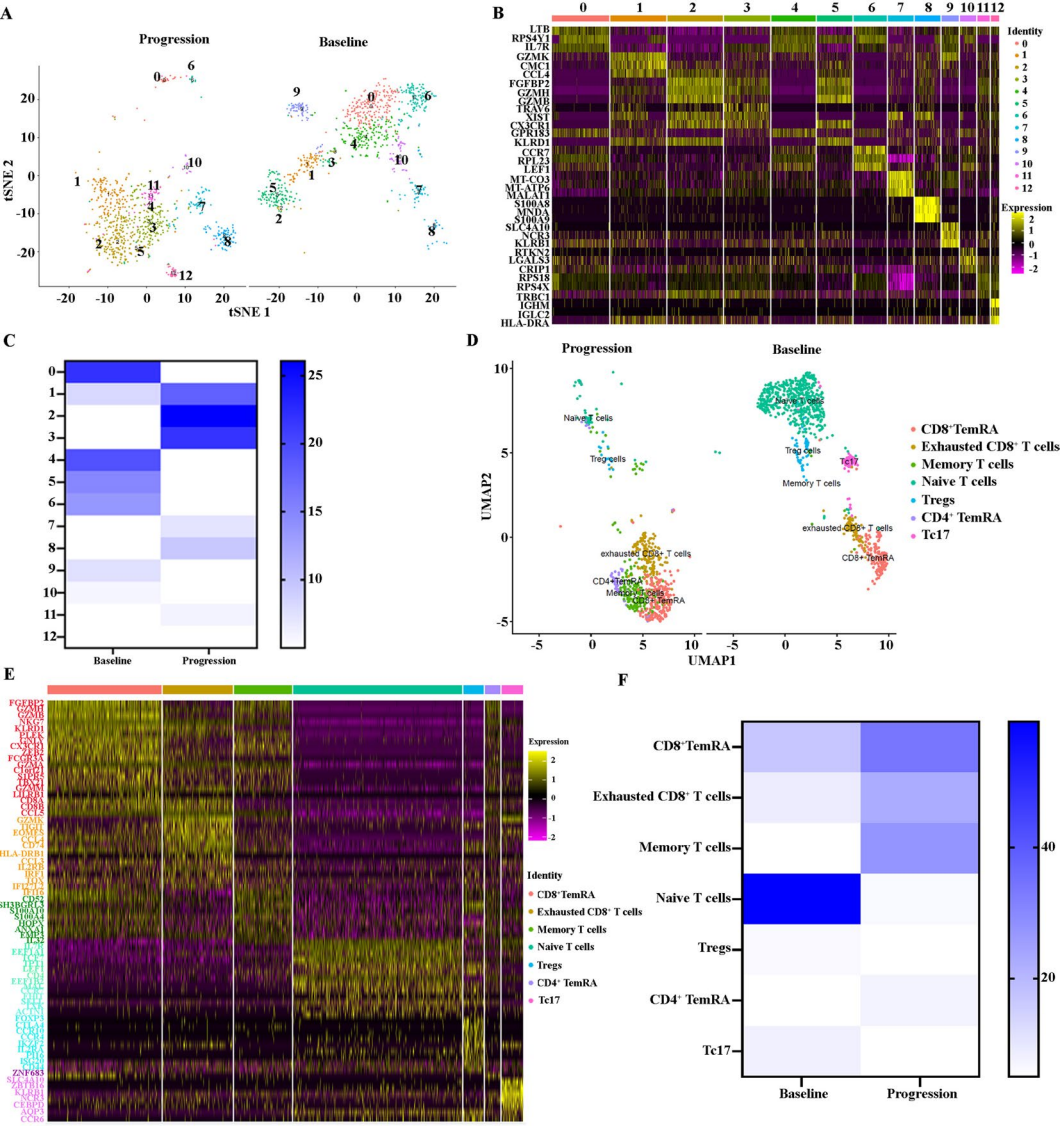
A significant increase in exhausted T cells at relapse after BCMA CAR-T cell therapy in R/R-MM. **A** The tSNE plots of T cells at baseline and progression. **B** The heatmap of differentially expressed genes in each cluster. **C** The proportions of T cells of all the clusters at baseline and progression. **D** The UMAP plots of CD8^+^TemRA, exhausted CD8^+^T cells, memory T cells, Naive T cells, Tregs, CD4^+^TemRA, and Tc17 at baseline and progression. **E** The heatmap of differentially expressed genes of CD8^+^TemRA, exhausted CD8^+^T cells, memory T cells, Naive T cells, Tregs, CD4^+^TemRA, and Tc17. **F** The proportion of CD8^+^TemRA, exhausted CD8^+^T cells, memory T cells, Naive T cells, Tregs, CD4^+^TemRA, and Tc17 at baseline and progression

We then analyzed the heterogeneity of the patients’ T cells at baseline and progression. The T cells exhibited 13 distinct subclusters (Fig. 3A). A heatmap of the top three marker genes for each cluster is shown in Fig. 3B. All marker genes of these 13 clusters are demonstrated in Additional file 3: Table S3. The percentages of each cluster at baseline and progression were 21.94% versus 3.64%, 8.20% versus 18.10%, 0.75% versus 26.09%, 0.64% versus 21.74%, 19.28% versus 0.24%, 14.91% versus 0.82%, 13.53% versus 1.18%, 4.79% versus 7.17%, 2.66% versus 9.52%, 7.56% versus 0%, 5.75% versus 1.53%, 0% versus 5.99%, and 0% versus 4.00%, respectively (Fig. 3C**)** The results show that the proportion of subpopulations 1, 2, 3, 7, 8, 11, and 12 at progression was elevated compared to the proportion of each subpopulation of T cells at baseline. In contrast, the proportion of subpopulations 0, 4, 5, 6, 9, and 10 at progression reduced compared to the proportion of each subpopulation of T cells at baseline. Interestingly, the proportion of clusters 1, 2 and 3 at progression was 65.93% and occupied the majority of the T-cell subpopulations. According to their DEGs, T cell subclusters were designated as Cluster 0: Naïve T cells (marked with EEF1A1, TPT1, IL7R,NOSIP,TIMP1,LTB, FLT3LG, and TCF7); Cluster 1: exhausted CD8^+^ T cells (marked with GZMK, TIGIT, EOMES, CCL4, CCL5, CD74, HLA-DRB1, CCL3, GZMA, IL2RB, IRF1, TOX, IFI27L2, and IFI16); Cluster 2: CD8^+^ TemRA (marked with FGFBP2, GZMH, GZMB, NKG7, KLRD1,PLEK, GNLY, CX3CR1, ZEB2, FCGR3A, GZMA, C1orf21,S1PR5, TBX21, and GZMM); Cluster 3: memory T cells (marked with CD52, SH3BGRL3, CX3CR1, S100A10, S100A4, HOPX, ANXA1, EMP3, GZMA, IL32, and CCL5); Cluster 4: Naïve T cells (marked with IL7R, EEF1A1, TCF7, TPT1, LEF1, CD4, EEF1B2, and MAL); Cluster 5: CD8^+^ TemRA (marked with GZMB, FCGR3A, KLRD1, FGFBP2, NKG7, CX3CR1, GNLY, GZMH, LILRB1, GZMH, CD8A, CD8B, ZEB2, S1PR5, GZMA, CCL5, TBX21, GZMM, and C1orf21); Cluster 6: Naïve T cells (marked with CCR7, MAL, FHIT, LEF1, TPT1, EEF1A1, TCF7, SELL, EEF1B2, TXK, and ACTN1); Cluster 7: uncharacterized (marked with NEAT1, N4BP2L2, PTPRC, BCL11B, DDX17, RIPOR2, and ARGLU1); Cluster 8: double cells; Cluster 9: Tc17 (marked with SLC4A10, ZBTB16, KLRB1, NCR3, CEBPD, IL7R, AQP3, and CCR6), Cluster 10: Treg (marked with FOXP3, CTLA4, CCR10, CCR4, IKZF2, IL2RA, CD4,PI16, ISG20, and CD44); Cluster 11: CD4^+^TemRA (marked with ZNF683, GZMH, and FGFBP2); Cluster 12: double cells (Fig. 3D and E). We then calculated the proportion of CD8^+^TemRA, exhausted CD8^+^T cells, memory T cells, Naive T cells, Tregs, CD4^+^TemRA, and Tc17 at baseline and progression. Significantly, the percentage of CD8^+^TemRA, exhausted CD8^+^T cells, memory T cells, and CD4^+^TemRA increased at progression. However, the proportion of Naive T cells, Tregs, and Tc17 decreased at progression (Fig. 3F). Taken together, the results reveal that T cells at progression display high immunosuppressive characteristics and exhausted status, which may contribute to the relapse after BCMA CAR-T cell therapy in R/R-MM.

### Significant increase in TIGIT^+^ NK cells at relapse after BCMA CAR-T cell therapy in R/R-MM

**Fig. 4:**
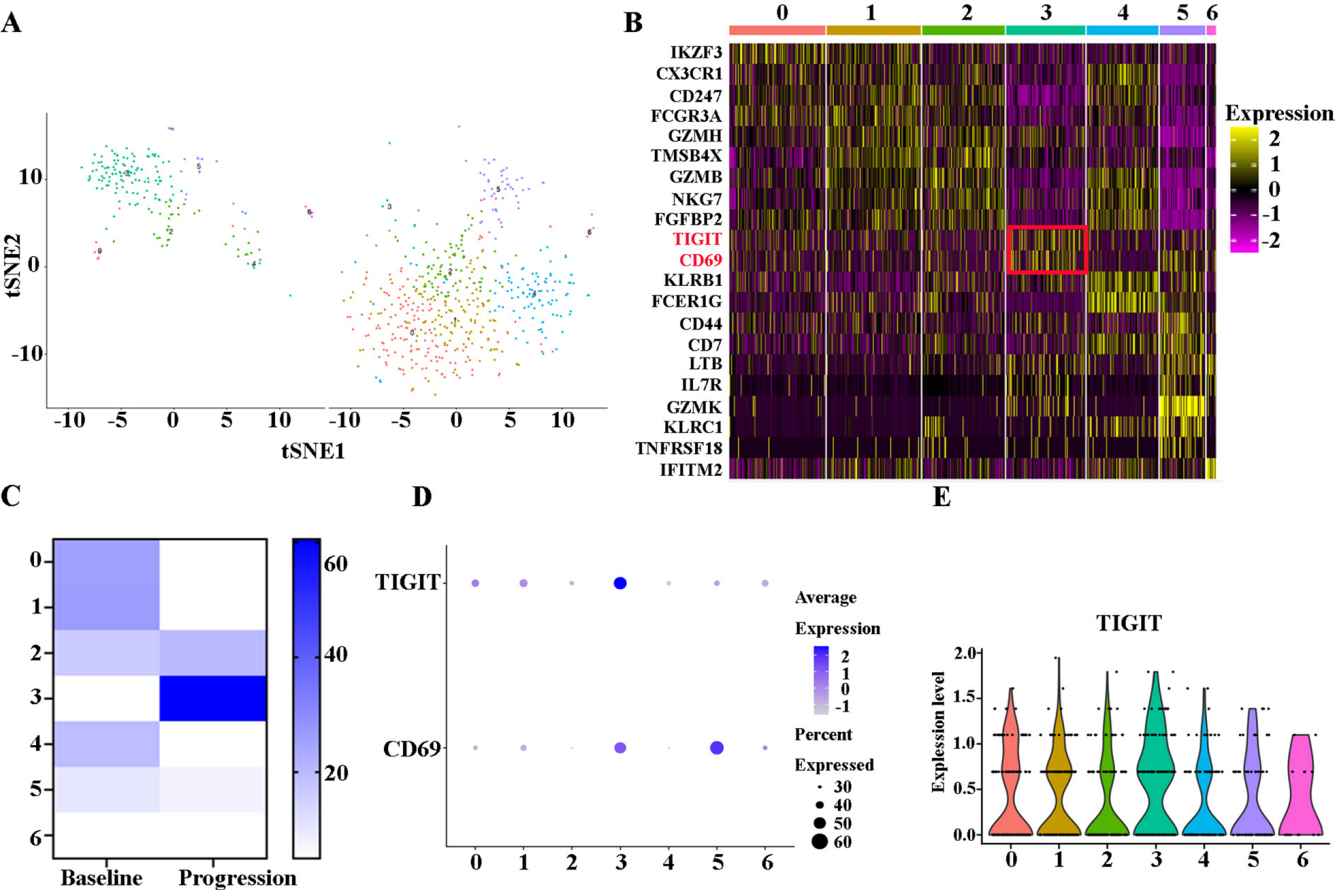
A significant increase in TIGIT^+^ NK cells at relapse after BCMA CAR-T cell therapy in R/R-MM. **A** The tSNE plots of NK cells at baseline and progression. **B** The heatmap of differentially expressed genes in each cluster. **C** The proportions of NK cells of all the clusters at baseline and progression. **D** Dot plot of TIGIT and CD69 expression in each cluster. **E** The violin diagram of TIGIT expression in each cluster

Natural killer (NK) cells are large granular lymphocytes that are involved in our defense against certain virus-infected and malignant cells [21]. We analyzed the heterogeneity of NK cells in the patients at baseline and progression. Seven subclusters were identified in all NK cells (Fig. 4A). A heatmap of the marker genes for each cluster is shown in Fig. 4B. All marker genes of these seven clusters are demonstrated in Additional file 4: Table S4. Thereafter, we calculated the change in the proportion of each subcluster. We found that the patient showed increases in Clusters 2, 3, and 6 at progression. Importantly, the proportion of Cluster 3 increased from 0.96% to 60.54% from baseline to progression (Fig. 4C). According to their DEGs, NK cell subclusters exhibited highly distinct marker genes: Cluster 0: IGF2R, PTPRC, and IKZF3; Cluster 1 (CD16^hi^ NK cells): CX3CR1, CD247, KIR2DL3, FCGR3A, GZMH, and TMSB4X; Cluster 2 (GZMB^hi^ NK cells): GZMB, NKG7, GZMA, FGFBP2, and TMSB4X; Cluster 3(TIGIT^hi^ NK cells): TIGIT and CD69; Cluster 4 (NKG7^hi^ NK cells): NKG7, GZMB, CX3CR1, KLRB1, and FCER1G; Cluster 5 (CD44^hi^ NK cells): CD44, CD7, LTB, IL7R, GZMK, KLRC1, and TNFRSF18; Cluster 6(Interferon-responsive NK cells): IFITM2 (Fig. 4B, D and E). In conclusion, the patient who relapsed after treatment with BCMA CAR-T cell therapy showed an increase in TIGIT^hi^ NK cells.

### The proportion of interferon-responsive DC cells increased significantly at relapse after BCMA CAR-T cell therapy in R/R-MM

In this study, 56 DC cells from baseline and progression were divided into three subclusters (Additional file 9: Fig. S2A). A heatmap of the marker genes for each cluster is shown in Additional file 9: Fig. S2B. All the marker genes of these three clusters are demonstrated in Additional file 5: Table S5. Interestingly, the proportion of Cluster 1 increased from 3.57% at baseline to 60.71% at progression. However, the proportion of the other two clusters significantly decreased (Additional file 9: Fig. S2C). According to their DEGs, DC cell subclusters exhibited highly distinct marker genes: Cluster 0 (HLA-DRB5^hi^ DC): FGL2, TNFAIP2, and HLA-DRB5; Cluster 1 (Interferon-responsive DC cells): PKIB, TMSB10, DUSP1, HLA-DRB1, HLA-DPA1, HLA-DRA, TIMP1, CD52, ITGB2, IFI30, CD83, TPI1, IFITM3, HLA-DQA1, LY86, HLA-DPB1, and ISG15; Cluster 2 (CLEC4C^hi^ LILRA4^hi^ DCs): TPM2, PTCRA, CLEC4C, IL3A, SCT, LRRC26, TLR9, and SPIB (Additional file 9: Fig. S2B, D, and E). Collectively, the proportion of interferon-responsive DC cells increased significantly in R/R-MM at progression.

### Interferon-responsive neutrophils increased significantly at relapse after BCMA CAR-T cell therapy in R/R-MM

**Fig. 5:**
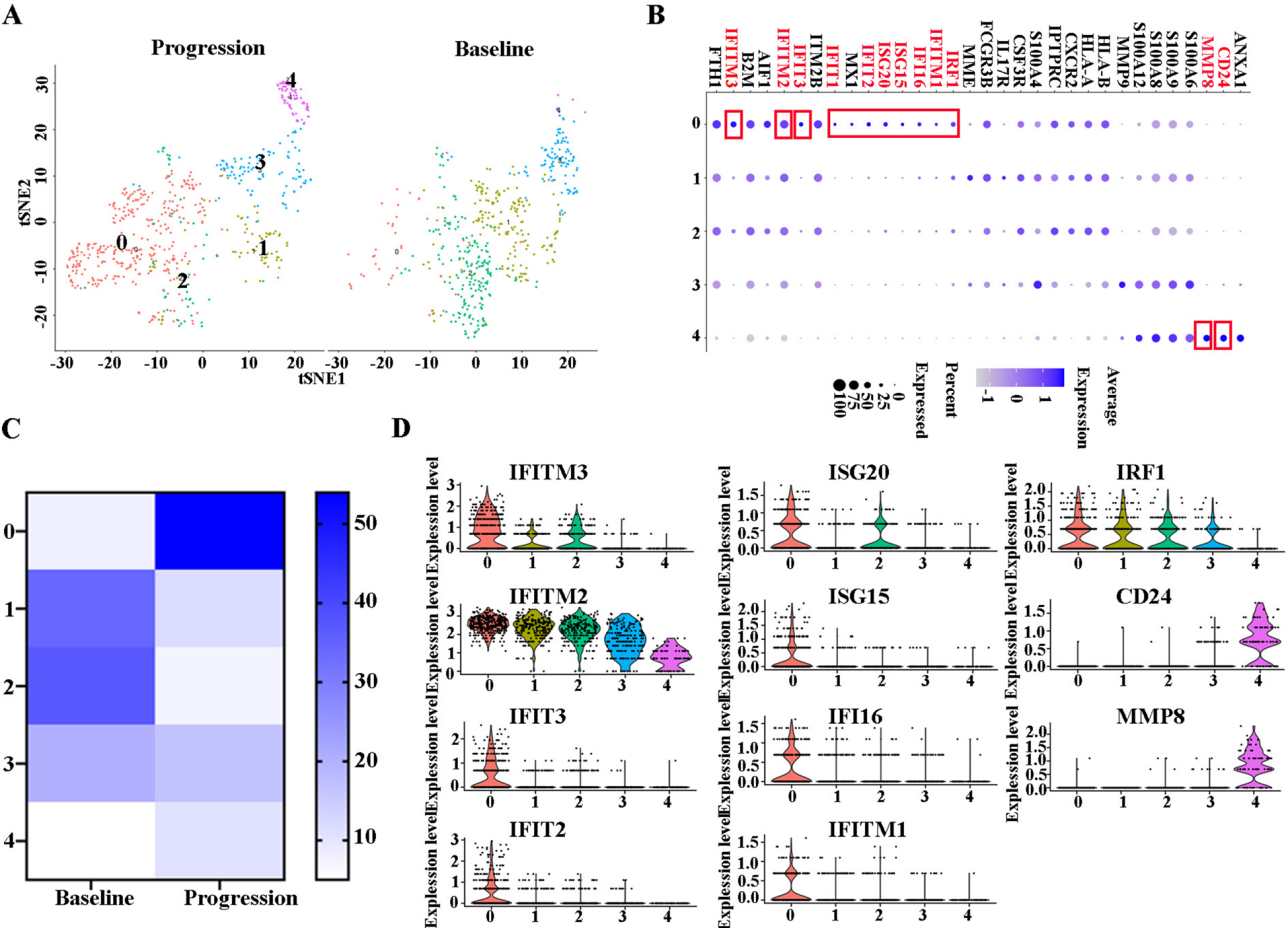
Interferon-induced neutrophils increases significantly at relapse after BCMA CAR-T cell therapy in R/R-MM. **A** The tSNE plots of neutrophils at baseline and progression. **B** Dot plot of differentially expressed genes in each cluster. **C** The proportions of neutrophils of all the clusters at baseline and progression. **D** The violin diagram of IFITM3, IFITM2, IFIT3, IFIT2, ISG20, ISG15, IFI16, IFITM1, IRF1, CD24, and MMP8 expression in each cluster

In this study, we identified five main subclusters following the reclustering of tumor-associated neutrophils (Fig. 5A). A FeaturePlot of the marker genes of each cluster is shown in Fig. 5B. All the marker genes of these seven clusters are demonstrated in Additional file 6: Table S6. Interestingly, the percentages of Clusters 0 and 4 were 53.92% and 10.72%, respectively, at progression, compared to 8.02%, and1.20% at baseline, respectively (Fig. 5C). According to their DEGs, DC cell subclusters exhibited highly distinct marker genes: Cluster 0 (Interferon-responsive neutrophils): FTH1, IFITM3, B2M, AIF1, IFITM2, IFIT3, ITM2B, IFIT1, MX1, IFIT2, ISG20, ISG15, IFI16, IFITM1, and IRF1; Cluster 1(FCGR3B^hi^ neutrophils): MME, FCGR3B, IL17RA, CSF3R, and S100A4; Cluster 2(CXCR2^hi^ neutrophils): PTPRC, CXCR2, HLA-A, and HLA-B; Cluster 3 (S100A12^hi^ neutrophils): S100A12, S100A8, S100A9, S100A4, and S100A6; Cluster 4(MMP8^hi^ neutrophils): MMP8, CD24, and ANXA1 (Figs. 5B and D). Cluster 0 highly expressed many interferon-induced genes and Cluster 4 expressed tumor-promoted genes. Collectively, the proportion of interferon-responsive neutrophils increased significantly in the R/R-MM patient at progression.

### Monocytes/macrophages display tumor-promoting phenotypes and induced exhausted T cells at relapse after BCMA CAR-T cell therapy in R/R-MM

**Fig. 6:**
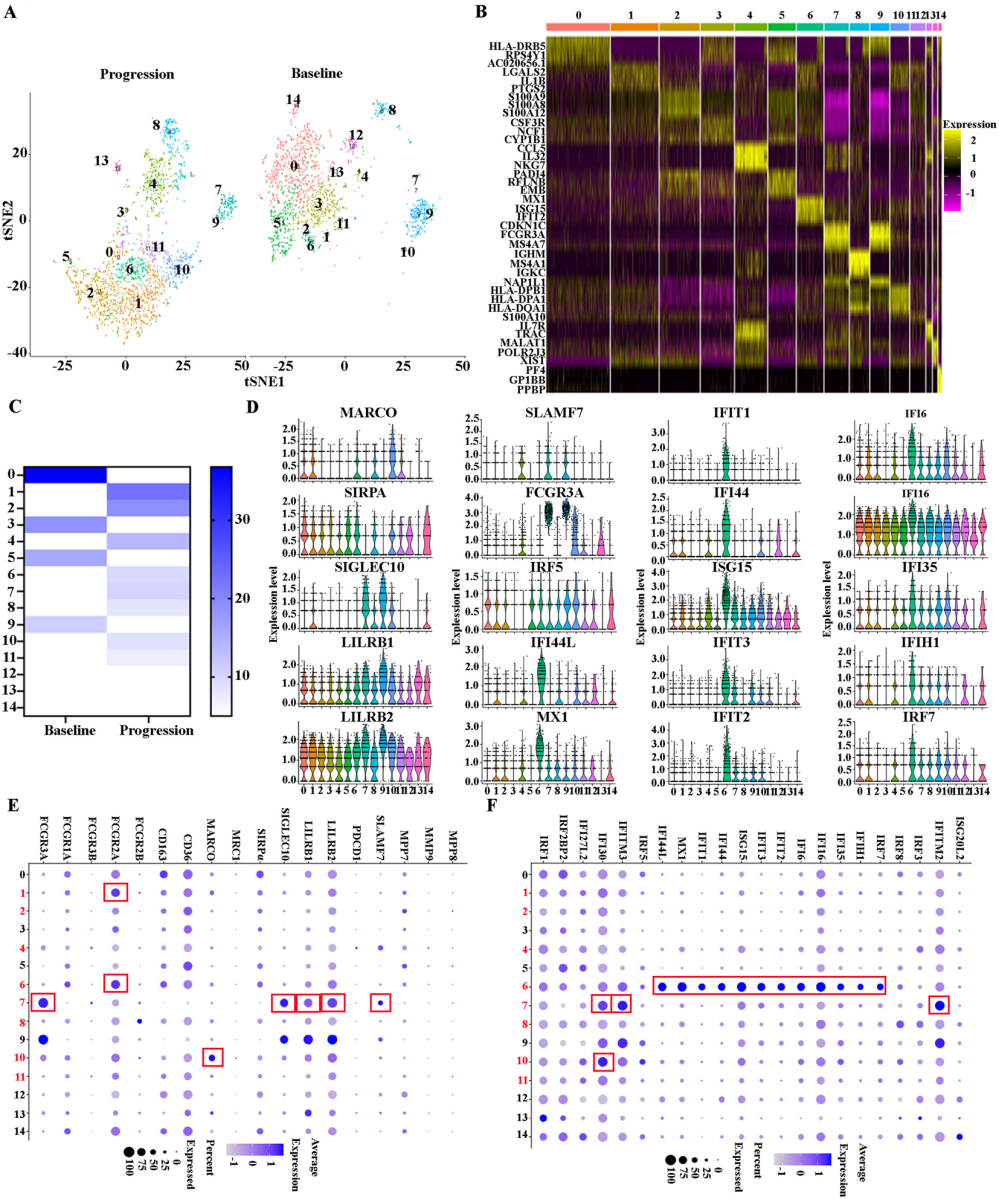
Monocytes/macrophages display tumor-promoting phenotypes and induced exhausted T cells at relapse after BCMA CAR-T cell therapy in R/R-MM. **A** The tSNE plots of monocytes/macrophages at baseline and progression. **B** The heatmap of differentially expressed genes in each cluster. **C** proportions of monocytes/macrophages of all the clusters at baseline and progression. **D–F** Dot plots of differentially expressed genes in each cluster

To better understand the heterogeneity of monocytes and macrophages at baseline and progression, we extracted and clustered these cells (1736 cells VS 2117 cells) by tSNE and obtained 15 subclusters (Fig. 6A). A heatmap of the top three marker genes for each cluster is shown in Fig. 6B. All the marker genes of these 15 clusters are demonstrated in Additional file 7: Table S7. The percentages of each cluster at baseline and progression were 36.81% versus 0.094%, 0.46% versus 22.44%, 0.29% versus 18.89%, 18.49% versus 0.71%, 1.73% versus 13.93%, 15.73% versus 0.28%, 3.69% versus 9.68%, 1.67% versus 10.30%, 4.03% versus 8.31%, 11.00% versus 0.05%, 0.29% versus 8.79%, 0.12% versus 7.13%, 3.23% versus 0%, 0.46% versus 1.75%, and 2.02% versus 0%, respectively (Fig. 6C). This result indicates that the proportion of Clusters 1, 2, 4, 6, 7, 8, 10, 11, and 13 increased in the patient at progression compared with baseline, and Clusters 1, 2, 4, 6, 7, 8, 10, and 11 occupied more than 80% of the total monocyte/macrophages at progression. Based on the DEGs, monocyte/macrophage subclusters exhibited highly distinct marker genes: Cluster 0: HLA-DRB5, CD74, HLA-A, HLA-DQB1, IRF2BP2, and SIRPα; Cluster 1(IL1β^hi^ Mφ): IL1β, CD14, HLA-DRB1, IFI30, FCGR2A, S100A10, S100A11, and MARCO; Cluster 2(S100A9^hi^ Mφ): S100A9, S100A8, S100A12, and HIF1A; Cluster 3: CSF3R and MNDA; Cluster 4: double cells; Cluster 5: IGFBP2, PADI4, RNASE2, and SLC40A1; Cluster 6 (Interferon-responsive Mφ): IFIT1, IFI44L, MX1, ISG15, IFIT3, IFIT2, IFI44, IFI6, STAT1, IFI16, IFITM3, IFIH1, IFI35, FCGR2A, IFIT5, and CD163; Cluster 7 (CD16^hi^ Mφ): FCGR3A, SIGLEC10, IFITM3, IFITM2, AIF1,HMOX1, C1QA, IFI30, SLAMF7, LILRB1, IFIT3, IFITM1, and ISG15; Cluster 8: double cells; Cluster 9: FCGR3A, SIGLEC10, CSF1R, IFITM3, IFITM2, HMOX1, LILRB1, AIF1, LILRB2, IFI30, CD68, and IFITM1; Cluster 10 (MARCO ^hi^ Mφ): HLA-DPB1, HLA-DPA1, HLA-DRB1, HLA-DRA, CD74, IFI30, MARCO, IFITM3, and ISG15; Cluster 11 (S100A11^hi^ Mφ): S100A11,S100A6, S100A4 and S100A10; Cluster 12: double cells; Cluster 13: CD74 and HLA-DQB1; Cluster 14: PF4 and HLA-DRB5 (Figs. 6D, E and F, Additional file 10: Fig. S3). Monocyte/macrophage expressed several markers, including Fc receptors [22, 23], scavenger receptors [24–28], “don’t eat me” genes [29, 30], MMP families [31], interferon-induced genes [32, 33], S100 families [34], cytokines [35], engulfment-related and iron recycling genes [36], and MHC molecules [37–39], which promote or inhibit the proliferation of tumors. We reanalyzed these markers, based on this category. Notably, at progression, the patient demonstrated enhanced Fc receptors, scavenger receptors, “don't eat me” signaling genes, and interferon-induced signaling markers (Fig. 6D, E and F). Finally, we analyzed the enrichment of the KEGG pathway in each cluster. The KEGG pathway in Cluster 1 included a Toll-like receptor signaling pathway, lipid and atherosclerosis, and an NF-kappa B signaling pathway; in Cluster 2, the KEGG pathway included drug metabolism—other enzymes, complement and coagulation cascades, and IL-17 signaling pathway; in Cluster 4, the KEGG pathway included a T cell signaling pathway, Th1 and Th2 cell differentiation, Th17 cell differentiation, natural killer cell mediated cytotoxicity, primary immunodeficiency, PD-L1 expression, and a PD-1 checkpoint pathway in cancer, and hematopoietic cell lineage; in Cluster 6, the KEGG pathway included a NOD-like receptor signaling pathway and leishmaniasis; In Cluster 7, the KEGG pathway included a B cell receptor signaling pathway and natural killer cell-mediated cytotoxicity; in Cluster 8, the KEGG pathway included antigen processing and presentation, graft-versus-host disease, and allograft rejection; and, in Cluster 10, the KEGG pathway included chagas disease, human cytomegalovirus infection, and influenza A (Additional file 11: Fig. S4). Taken together, monocytes/macrophages displayed tumor-promoting phenotypes and induced exhausted T cells in the R/R-MM patient at progression.

### Cell–cell communication analysis demonstrated monocytes/macrophages are key player at relapse after BCMA CAR-T cell therapy in R/R-MM

To address the cell–cell communication at relapse after BCMA CAR-T cell therapy in R/R-MM, we performed communication patterns analysis as previous described [19, 40–43]. The communication patterns that connect cell groups with signaling pathways either in the context of outgoing signaling (i.e., treating cells as senders) or incoming signaling (i.e., treating cells as receivers). Firstly, this analysis uncovered four patterns for outgoing signaling (Additional file 12: Fig. S5A). Significantly, a large portion of outgoing monocyte/macrophage signaling is characterized by pattern #2 (Additional file 12: Fig. S5A and B), which represents multiple pathways, including but not limited to BAG, RESISTIN, MIF, ITGB2, APRIL, and MPZ (Additional file 12: Fig. S5A and C). Next, we found four patterns for incoming signaling (Additional file 12: Fig. S5D). The large portion of incoming monocyte/macrophage signaling is characterized by pattern #1 (Additional file 12: Fig. S5D and E), which represents multiple pathways, including but not limited to GRN, PECAM1, ANNEXIN, CSF, CXCL, and CCL (Additional file 12: Fig. S5D and F). Receptor-ligand analysis of different cell subpopulations also revealed that the most frequent interaction with other cell types was with monocytes/macrophages (Additional file 12: Fig. S5G).

**Fig. 7:**
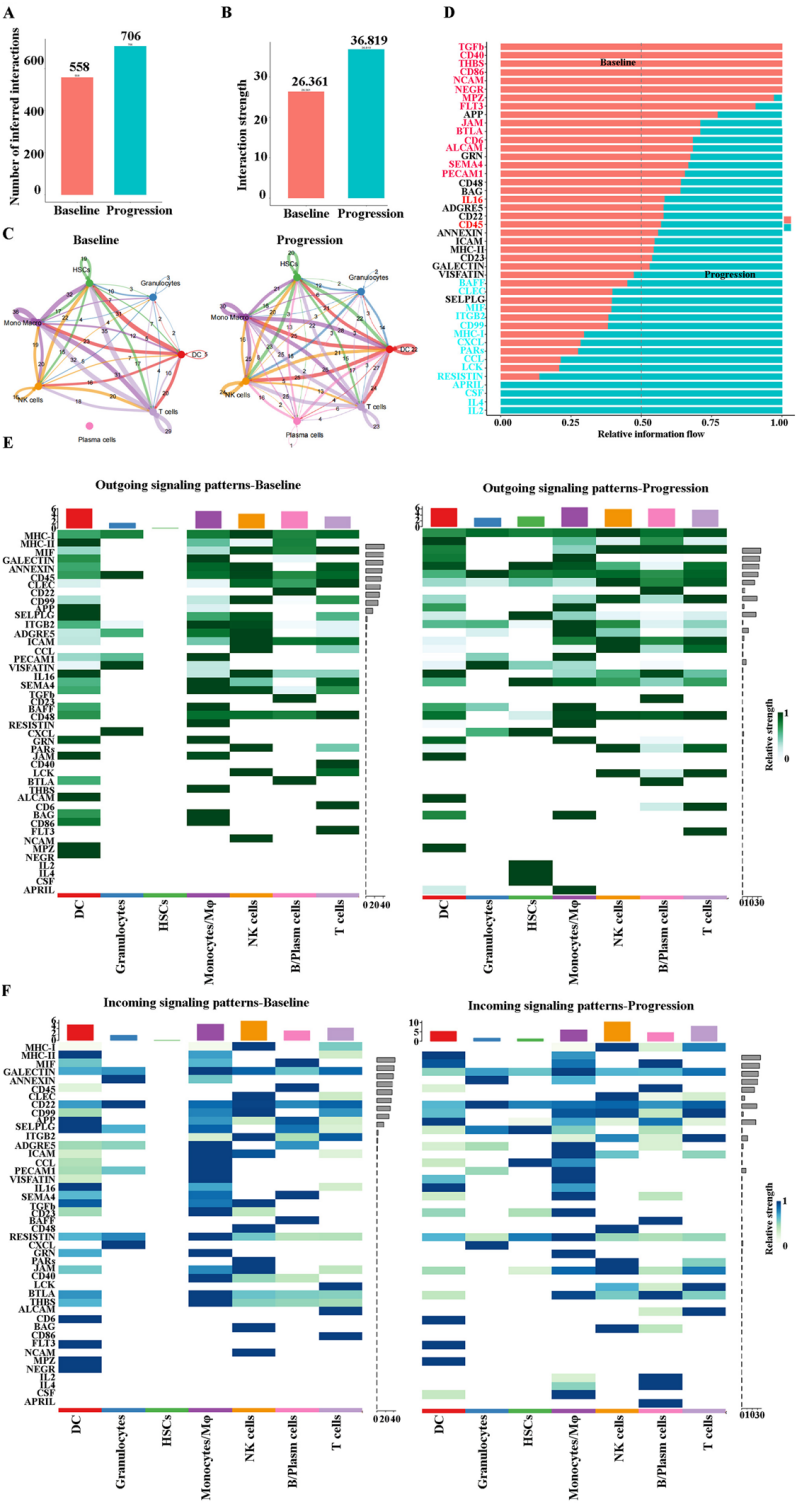
The difference of signaling pathways between baseline and progression in R/R-MM. **A** The number of inferred interactions between baseline and progression in R/R-MM. **B** The interaction strength between baseline and progression in R/R-MM. **C** The number of inferred interactions for each group. **D** The overall information flow of each signaling pathway between baseline and progression in R/R-MM. **E** The outgoing signaling of each cell population between baseline and progression in R/R-MM. **F** The incoming signaling of each cell population between baseline and progression in R/R-MM

Next, we examined the difference of signaling pathways between baseline and progression in R/R-MM. Figure 7A and B demonstrated that both the number of inferred interactions and interaction strength increased at progression in R/R-MM. Significantly, the interactions between plasma cells and other cell types presented in this study also increased at progression in R/R-MM (Fig. 7C). Then, we analyzed the overall signaling pathways of each cell population between baseline and progression in R/R-MM. Figure 7D indicated that the signaling pathways at progression in R/R-MM included BAFF, CLEC, MIF, ITGB2, CD99, MHC-I, CXCL, PARs, CCL, LCK, RESISTIN, APRIL, CSF, IL4 and IL2. The outgoing signaling patterns of monocyte/macrophage at progression included APRIL, MIF, RESISTIN, BAFF, ITGB2, CLEC, MHC-I, and CD99 (Fig. 7E). And the incoming signaling patterns of monocyte/macrophage at progression included MIF, CD99, ITGB2, CCL, CSF, IL4, MHC-I, and IL2 (Fig. 7F). Collectively, our study suggests that monocytes/macrophages may play an important role in R/R-MM at relapse after BCMA CAR-T cell therapy.

## Discussion

Recent domestic and international studies have reported significant efficacy of BCMA CAR-T in R/R-MM, with some reports of even achieving ORR of 100%. Idecabtagene vicleucel (BCMA CAR-T) was approved by the FDA on March 26, 2021. Despite these promising clinical outcomes of anti-BCMA CAR T-cell therapy in R/R- MM, some subsets of patients are still at risk of relapse during this treatment, which has become another focus of attention. Therefore, there is an urgent need to explore the role of myeloma self-factors as well as BM microenvironment factors at relapse after BCMA CAR-T cell therapy. In this study, we reported a case initial response followed by the development of a resistant phenotype, as represented in a relapse after BCMA CAR-T treatment. ScRNAseq analyses of CD45^+^ cells were performed before the initiation of BCMA CAR-T therapy and at relapse after BCMA CAR-T therapy. Interestingly, the patient had a higher proportion of monocytes/macrophages and a lower proportion of T cells at relapse, which is consistent with the report from an ASH meeting in 2021 [44]. In their report, *David T. Melnekoff* mentioned that they found that those who responded well to BCMA CAR-T immunotherapy had a higher proportion of CD8^+^ T cells and a lower proportion of CD14^+^ monocytes. Our and other previous studies have demonstrated that T cells and monocytes/macrophages may contribute to relapse after BCMA CAR-T cell therapy.

Previous studies have indicated that BCMA represents an important component of plasma cell function, and, thus, its loss is not frequently observed [17, 45]. Indeed, the loss of BCMA after BCMA CAR-T cell therapy has previously been reported only on a case-by-case basis in the literature [17, 18]. In the present study, we found that there are BCMA positive and BCMA negative plasma cells in the patient at relapse after BCMA CAR-T cell therapy. Studies have shown that MM usually demonstrates substantial inter- and intra-tumor heterogeneity, which is closely related to progression, resistance to therapy, and recurrences [46–50]. Our study also confirms that plasma cells with heterogeneous changes emerged. At baseline, there are approximately 21.8% plasma cells with BCMA, CD38, CD24, CD138, SLAMF7, and GPRC5D expression; however, 51.8% of these plasma cells emerged at progression. Due to the highly heterogeneous nature of R/R-MM, relapse after BCMA CAR-T cell therapy is a difficult clinical problem, and there is a clinical need to find more targets for patients who relapse after BCMA CAR-T cell therapy. Based on this study, potential targets that could be selected for the preparation of new CAR-T cells include CD38, CD24, CD138, SLAMF7, and GPRC5D. *Esther Drent* et al. reported that CD38 CAR-T cells have time- and dose-dependent killing effects on MM cells in vitro and have also shown important anti-tumor effects in a xenotransplant mouse model [51]. A clinical trial of CAR-T cells targeting CD38 for R/R-MM is currently underway (NCT03464916). Furthermore, CAR-T Cells that target both BCMA and CD38 for R/R-MM have demonstrated strong cytotoxic activity in clinical studies, with ORR up to 90.9% [52–54]. *FumouSun* et al. demonstrated that a BCMA-CD24 CAR-T yields strong cytotoxic activity and selectivity for MM cells in vitro and in vivo [55]*.* Future studies should be aimed at determining whether BCMA-CD24 CAR-T can show clinical efficacies in clinical trials. *BoGuo *et al. report that the treatment of CD138 CAR-T is safe, feasible, and tolerable and has potential anti-tumor activity in vivo, warranting further research into MM treatment with CD138 CAR-T [56]. In preclinical studies, SLAMF7 CAR-T cells showed strong anti-tumor effects against both MM cell lines and primary myeloma cells and prolonged the survival of mice with human MM.1S and IM9 myeloma cell xenografts [57]. However, since a certain amount of SLAMF7 is also expressed on the surface of T cells, it triggers T cell cross-reactivity in vivo, leading to self-killing of CAR-T cells [57]. In addition, researchers have developed a new SLAMF 7 CAR-T product, which specifically targets SLAMF7 in both in vitro and mouse models by knocking out the SLAMF7 gene from T cells prior to the introduction of SLAMF7 CAR, effectively killing MM cell lines and primary human myeloma cells [58]. Significantly, a phase-I/IIA clinical study of UCARTCS1 for the treatment of MM is ongoing (EudraCT: 2019–001,264-30). A head-to-head preclinical study of GPRC5D CAR-T versus BCMA CAR-T with the same CAR structure found that GPRC5D CAR-T showed comparable antigen-specific cytotoxicity to BCMA CAR-T cells in both ex vivo and in vitro experiments [59]. This study further demonstrated that GPRC5D-targeted CAR-T cell therapy can overcome tumor escape in a model of tumor recurrence due to BCMA antigen loss [59]. Preliminary clinical trial results have also shown that the first-in-class GPRC5D-targeted CAR T cell therapy for MM has a manageable safety profile with no serious or unexpected toxicities; this dose escalation study is ongoing with additional patients planned for treatment at higher doses. Efficacy is promising in intensively pre-treated R/R-MM, as reflected in high rates of clinical responses as well as in MRD-negativity, even at doses as low as 25 × 10^6^ CAR T cells. It is clinically important to note that all six patients who relapsed after BCMA CAR T therapy responded to GPRC5D-targeted CAR T therapy, including two patients who achieved sCR. In addition, preclinical studies as well as clinical trials of BCMA-based CAR-T cell therapy in combination with other targets have achieved good efficacy, including combined infusions of humanized BCMA CAR-T and CD19 CAR-T [60], BCMA and CD19 bispecific CAR-T cells [61], BCMA and CD38 dual-targeted CAR-T cells [52, 54], and BCMA and SLAMF7 bispecific CAR-T cells [62, 63]. More clinical trials should be conducted on patients who relapsed after BCMA CAR-T cell therapy to clarify the role of CAR-T cells with the above targets in these patients.

It is well known that T-cell exhaustion is an alteration of the differentiation spectrum that is characteristically undesirable for cancer immunotherapy [64]. T-cell exhaustion is also a mechanism for CAR-T cell therapy failure [20]. T cells composition (low CD4:CD8 ratio and reduced population of Tscm, Tcm) in MM patients, along with an enrichment of terminally exhausted T cells, are the main features observed in BCMA targeted CAR-T or BCMA-CD3 BiTEs therapies-resistant patients [65]. In pediatric B-ALL, B-cell recovery in peripheral blood within 3 months indicates a high risk of relapse with CD19 CAR-T cell therapy, which may be due to T-cell exhaustion [66]. A high percentage of LAG3^+^ T cells in relapsed/refractory diffuse large B cell lymphoma (R/R-DLBCL) patients is a biomarker of T cell exhaustion and correlates with lower ORR and higher recurrence rate after CD19 CAR-T cell therapy [67]. A study has indicated that CARs on CAR-T cells spontaneously aggregated antigen independently, causing CAR-CD3ζ signals to accumulate and lead to CAR-T cell exhaustion [68]. In addition, endogenous TCR signaling by CAR-T cells has been shown to induce T cell exhaustion in the presence of specific antigens [69]. In the present study, we demonstrated that the percentage of exhausted CD8^+^ effector T cells increased approximately 35-fold in the relapsed patients after BCMA CAR-T treatment, compared to the percentage at baseline. IFN-responding CD8^+^ effector T cells also increased significantly in the relapsed patients after BCMA CAR-T treatment, which also present exhausted phenotypes [70]. In conclusion, T cell exhaustion may play a major role in relapse after BCMA CAR-T cell treatment, and our data suggests that strategies to reduce exhaustion in BCMA CAR T cells will enhance clinical activity.

The majority of studies on the impact of the tumor microenvironment on immunotherapy have been performed with solid tumor patients, and data on hematological malignancies are quite limited [71]. However, CAR-T cell immunotherapy has been extremely successful, mainly for hematological tumors. One study has indicated that higher numbers of activated/functional T cells and lower numbers of CD163^+^macrophages prior to treatment correlated with a durable response to CD19 CAR-T therapy in R/R-DLBCL [72]. Another study analyzed initial tumor samples from patients with DLBCL treated with CAR-T cells and found that the macrophage content and expression of interferon signaling genes in immune cell subsets of the tumor microenvironment were associated with CAR-T cell treatment failure. High interferon signaling could induce high expressions of PD-L1 and MHC-II in tumor cells, which bind to PD-1 and LAG3 on the surface of T cells, respectively, and results in reduced CAR-T cell expansion and increased exhaustion [73]. One study has found that approximately 21.8% of MM patients treated with BCMA CAR-T cells developed macrophage activation syndrome-like (MAS-L) manifestations. Although there was no statistically significant difference in the overall treatment response rate, a comparison of the 1-year survival and progression-free survival time between the two groups of patients with and without MAS-L showed that the patients with MAS-L had lower overall treatment response rates, 1-year survival, and progression-free survival time than those without MAS-L [74]. These studies suggest that altered macrophage function may be an important microenvironmental factor in the short remission time and high relapse rate of patients treated with CAR-T cell therapy. However, little is known about how the BM microenvironment of R/R-MM patients changes at relapse after BCMA CAR-T cell therapy. In the present study, we found that the percentage of monocytes/macrophages increased at relapse after BCMA CAR-T cell therapy. Significantly, monocytes/macrophages display tumor-promoting phenotypes and induce exhausted T cells at relapse after BCMA CAR-T cell therapy in R/R-MM. Previous studies indicated that MARCO-expressing TAM numbers correlated with increased occurrence of regulatory T cells and effector T cells and decreased natural killer (NK) cells in tumors [28]. And targeting MARCO on macrophages within the tumor alters their polarization and in turn activate NK cells to kill the tumor [27]. In the present study, MARCO^hi^ monocytes/macrophages increased at relapse after BCMA CAR-T cell therapy in R/R-MM, suggesting targeting MARCO may also a novel treatment strategy for R/R-MM patients. S100 family expression is closely associated with tumor progression and poor prognosis [34]. Importantly, these molecules were highly expressed by Clusters 2 and 11, which showed an increased proportion in the patient at progression. Interferon-induced genes expressed by monocytes/macrophages yield the increase of exhausted T cells [70]. Interestingly, the increased proportion of Clusters 6, 7 and 10 at progression expressed several interferon-induced genes, which may induce the increase of exhausted T cells. These studies and our findings further confirmed that monocytes/macrophages display tumor-promoting phenotypes and induce immunosuppression to increase the percentage of exhausted T cells at relapse after BCMA CAR-T cell therapy in R/R-MM. Additionally, cell–cell communication analysis also demonstrated that monocytes/macrophages play an important role in the progression of R/R-MM at relapse after BCMA CAR-T cell therapy. The identified outgoing signaling pathways of monocytes/macrophages included APRIL, MIF, RESISTIN, BAFF, ITGB2, CLEC, and CD99. And the incoming signaling patterns of monocyte/macrophage at progression included MIF, CD99, ITGB2, CCL, CSF, IL4, and IL2. Previous studies indicated that APRIL expressed by monocytes/macrophages is a ligand of BCMA expressed by multiple myeloma cells, which can rescue interleukin 6 (IL-6)–dependent MM cell lines from apoptosis following IL-6 deprivation, stimulate MM cell growth via cyclin D-dependent G1/S cell cycle progression and induced immunosuppression in the bone marrow microenvironment [75–79]. Macrophage migration inhibitory factor (MIF) has been shown to promote disease progression directly in many malignancies, including multiple myeloma (MM) [80–82]. MIF also reshaped the macrophage phenotype to immunosuppressed status, which then promotes disease progression in MM [83]. Collectively, our study discovered critical cellular cross-talks between monocytes/macrophages, and plasma cells, which directly promotes the progression of MM. Additionally, monocytes/macrophages expressed APRIL and MIF may also contribute immunosuppression of BM microenvironment and then promotes the relapse of R/R-MM after BCMA CAR-T treatment. BCMA CAR-T cell therapy in combination with targeting macrophage therapeutics may be a novel treatment option to prolong the survival time of R/R-MM patients [30]. Further logical experimental verification should be conducted to confirm our findings. It would also be worthwhile to conduct clinical trials to confirm this hypothesis.

Other immune cells, such as NK cells [84], DC cells [85], and neutrophils [86], also affect the anti-tumor immune responses. However, the role of NK cells, DC cells, and neutrophils at relapse after BCMA CAR-T cell therapy is still unclear. Significantly, our study indicates that the proportions of TIGIT^+^ and/or CD69^+^ NK cells were significantly higher in patients who relapsed after BCMA CAR-T cell therapy, with an approximately 63-fold increase in the proportion, reaching up to 60%. TIGIT is a checkpoint receptor thought to be involved in mediating NK cell exhaustion in tumors [87]. Anti-CD69 mAb enhances the activity of host NK cells, mediated by a reduction in TGF-β [88]. These studies suggest that the inhibitory NK cell-mediated anti-tumor effect may contribute to a relapse after BCMA CAR T-cell treatment. DCs are key determinants in initiating and maintaining an effective T cell-mediated anti-tumor immune response. There is significant heterogeneity of DC cells in the tumor microenvironment, and different functional subtypes of DC cells may determine the efficacy of cancer immunotherapy [89]. Increased percentages of the ISG15^+^ DC subpopulation at progression were found in the present study. Studies have reported that ISG15 induces the expression of E-cadherin in DCs in vitro, and E-cadherin is an adhesion molecule; its expression can impair DC motility and act as a mechanism of melanoma tumor escape [90, 91]. Thus, our study suggests that the ISG15^+^ DC subpopulation may also contribute to the relapse after BCMA CAR T-cell treatment. Current studies have demonstrated that neutrophils can play the dual role of promoting tumor progression and inhibiting tumor proliferation [92]. Two clusters of neutrophils increased at progression. Cluster 0 expressed many interferon-induced genes, such as ISG15, IRF1, ISG20, and more, which play a major role in the development and metastatic progression of cancers [93, 94]. Cluster 4 expressed MMP8, which is mainly produced by neutrophils that can either inhibit or promote tumor progression in certain cancers [95–97]. Although the findings are controversial, the fact that neutrophils play a dual role by either promoting or suppressing cancer is undeniable. The plasticity of neutrophils enables them to adapt to different cancer microenvironments and to have varying effects on cancer. Further studies are also needed to define the roles of these two unique subpopulations of neutrophils in R/R-MM patients at relapse after BCMA CAR-T therapy.

However, this study has limitations. We only evaluated one patient at baseline and one patient at progression in the present study. A larger number of patients should be included in future studies and more studies should be also conducted to confirm our findings. Additionally, relapsed cases in the non-CAR-T group should also be included in the future studies. Nevertheless, our study established the potential existence of BCMA positive and negative relapse mechanisms at relapse after BCMA CAR-T cell therapy. Combination therapy aiming at additional MM targets (such as CD38, CD24, CD138, SLAMF7, and GPRC5D) may be a better CAR-T treatment strategy to overcome the heterogeneity of MM and avoid clonal selection based on the loss of one specific antigen. These approaches include bi-specific CAR-T products, trispecific antibody CAR-T products, and CAR-T products in combination with single-target immunotherapies. In addition, T-cell exhaustion is also one of the main reasons for the relapse after BCMA CAR-T cell therapy. Monocytes/macrophages may also promote progression of MM directly and induced immunosuppressive state to increase T cell exhaustion and suppress the NK cell function, and more, which then promote disease progression.

## Supplementary Information

**Additional file 1: Table S1.** The clinicopathological characteristics of the two R/R-MM patients in the present study.

**Additional file 2: Table S2.** All the marker genes of the 13 clusters in B/plasma cells.

**Additional file 3: Table S3.** All the marker genes of the 13 clusters in T cells.

**Additional file 4: Table S4.** All the marker genes of the 7 clusters in NK cells.

**Additional file 5: Table S5.** All the marker genes of the 3 clusters in DC cells.

**Additional file 6: Table S6.** All the marker genes of the 7 clusters in neutrophils.

**Additional file 7: Table S7.** All the marker genes of the 15 clusters in monocytes/macrophages.

**Additional file 8: Figure S1.** The heatmap of marker genes in each cell population

**Additional file 9: Figure S2.** The proportion of interferon-responsive DC cells increases significantly at relapse after BCMA CAR-T cell therapy in R/R-MM. **(A)** The tSNE plots of DC cells at baseline and progression. **(B)** The heatmap of differentially expressed genes in each cluster. **(C)** The proportions of DC cells of all the clusters at baseline and progression. **(D)** Dot ploFt of HLA-DPB1, HLA-DQA1, IFI30, IFITM3, and ISG15 expression in each cluster. **(E)** The violin diagram of IFI30, IFITM3, and ISG15 expression in each cluster.

**Additional file 10: Figure S3.** Dot plots of differentially expressed genes in each cluster.

**Additional file 11: Figure S4.** The enrichment of KEGG pathway in each cluster of monocytes/macrophages.

**Additional file 12: Figure S5.** The communication patterns that connect cell groups with signaling pathways either in the context of outgoing signaling or incoming signaling.

## Data Availability

All data generated and materials in the study are included in the present article and supplementary data.

## Abbreviations

BM: Bone marrow
DEGs: Differentially expressed genes
DLBCL: Diffuse Large B Cell Lymphoma
KEGG: Kyoto Encyclopedia of Genes and Genomes
MAS-L: Macrophage activation syndrome-like manifestation
NKs: Natural killer cells
ORR: Objective response rate
PFS: Progression-free survival time
R/R-MM: Relapsed/refractory multiple myeloma
scRNA-seq: Single-cell RNA sequencing
UMAP: Uniform manifold approximation and projection
