# Supplementary figures and images for: Identification of potential resistance mechanisms and therapeutic targets for the relapse of BCMA CAR-T therapy in relapsed/refractory multiple myeloma through single-cell sequencing
Source: Exp Hematol Oncol. 2023 May 8;12:44. doi: 10.1186/s40164-023-00402-5 (PMC10165782; doi:10.1186/s40164-023-00402-5)

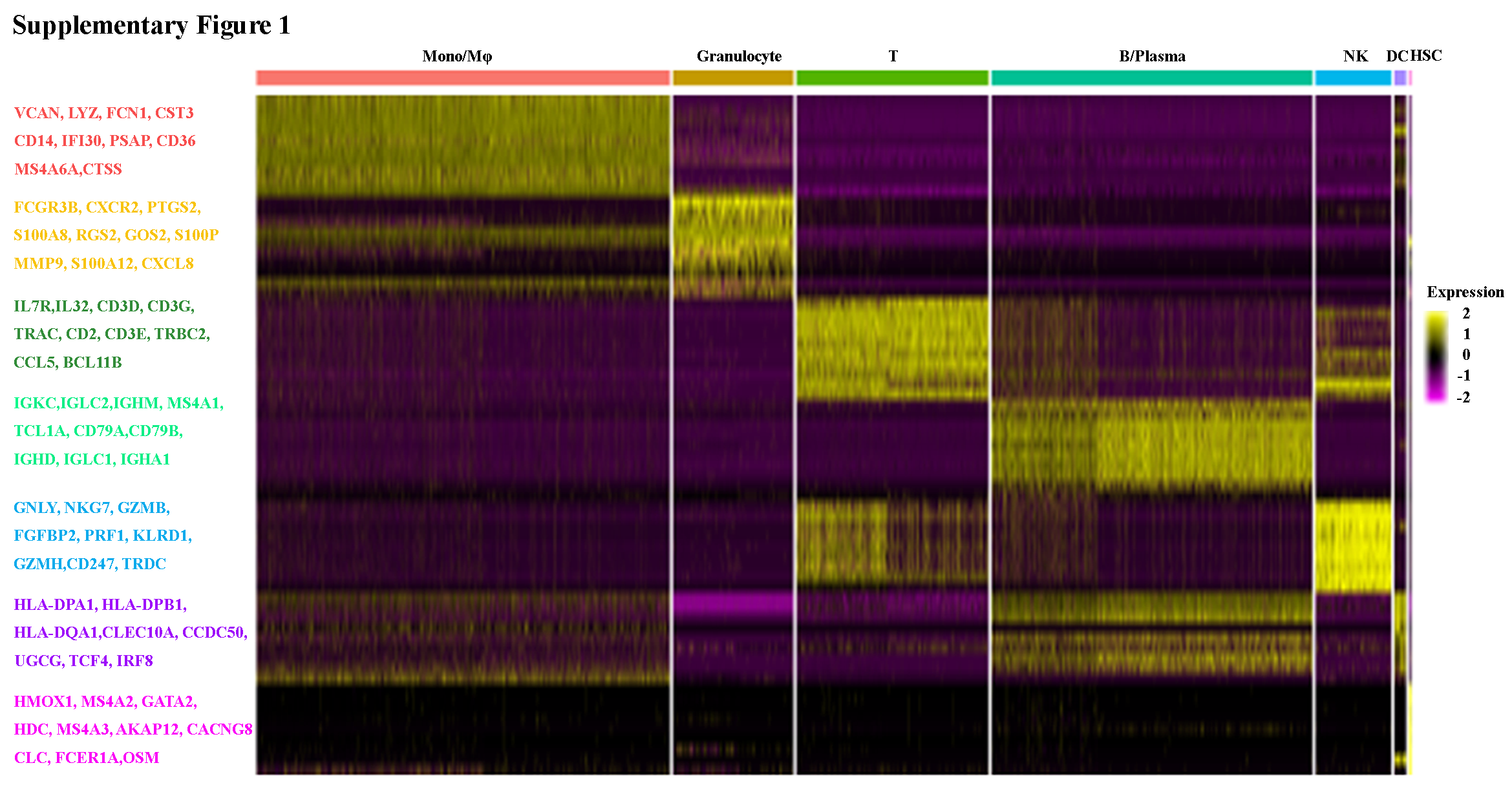

Supplement: Supplementary file 8 — Additional file 8: Figure S1. The heatmap of marker genes in each cell population [file 40164_2023_402_MOESM8_ESM.tif]

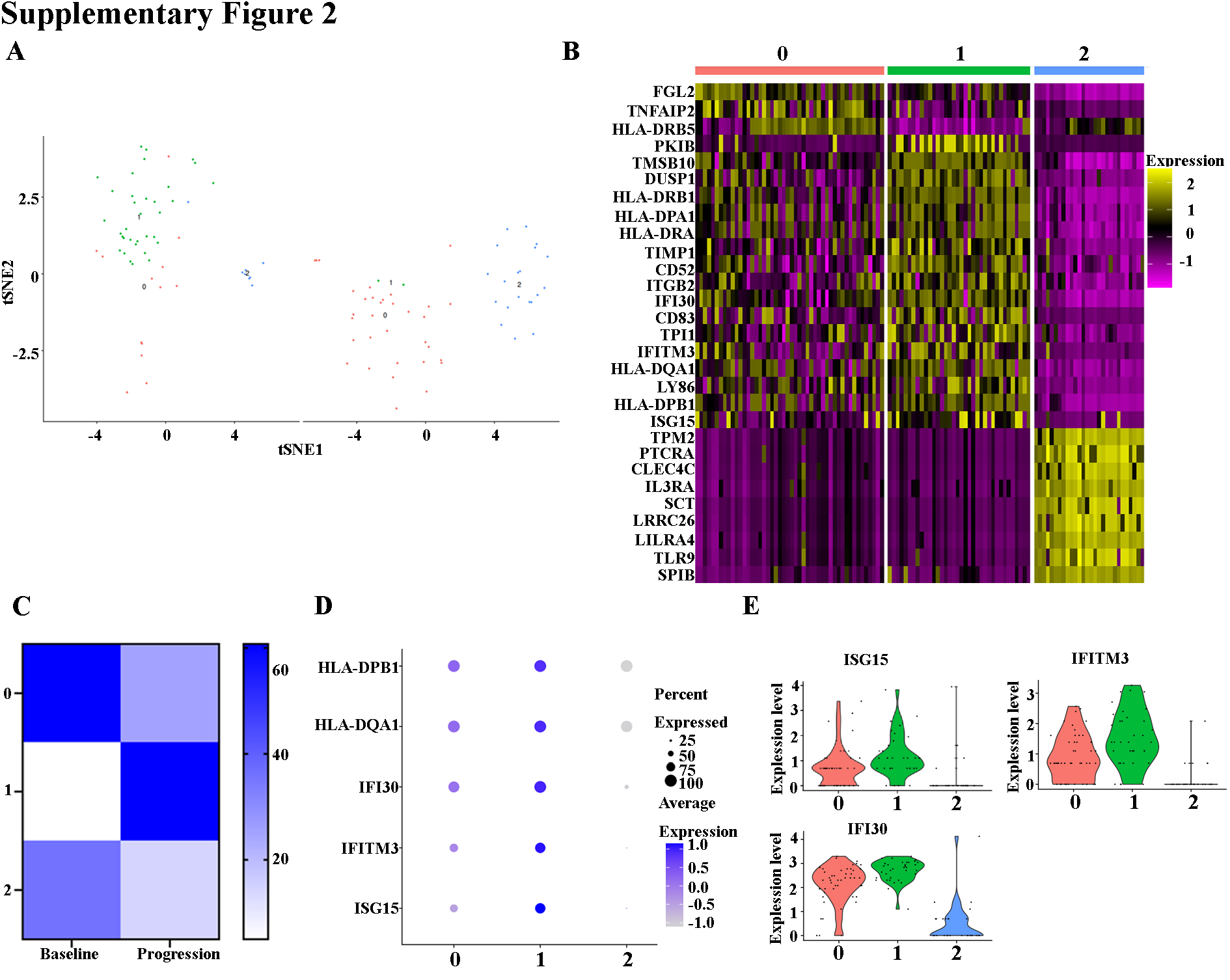

Supplement: Supplementary file 9 — Additional file 9: Figure S2. The proportion of interferon-responsive DC cells increases significantly at relapse after BCMA CAR-T cell therapy in R/R-MM. (A) The tSNE plots of DC cells at baseline and progression. (B) The heatmap of differentially expressed genes in each cluster. (C) The proportions of DC cells of all the clusters at baseline and progression. (D) Dot ploFt of HLA-DPB1, HLA-DQA1, IFI30, IFITM3, and ISG15 expression in each cluster. (E) The violin diagram of IFI30, IFITM3, and ISG15 expression in each cluster. [file 40164_2023_402_MOESM9_ESM.tif]

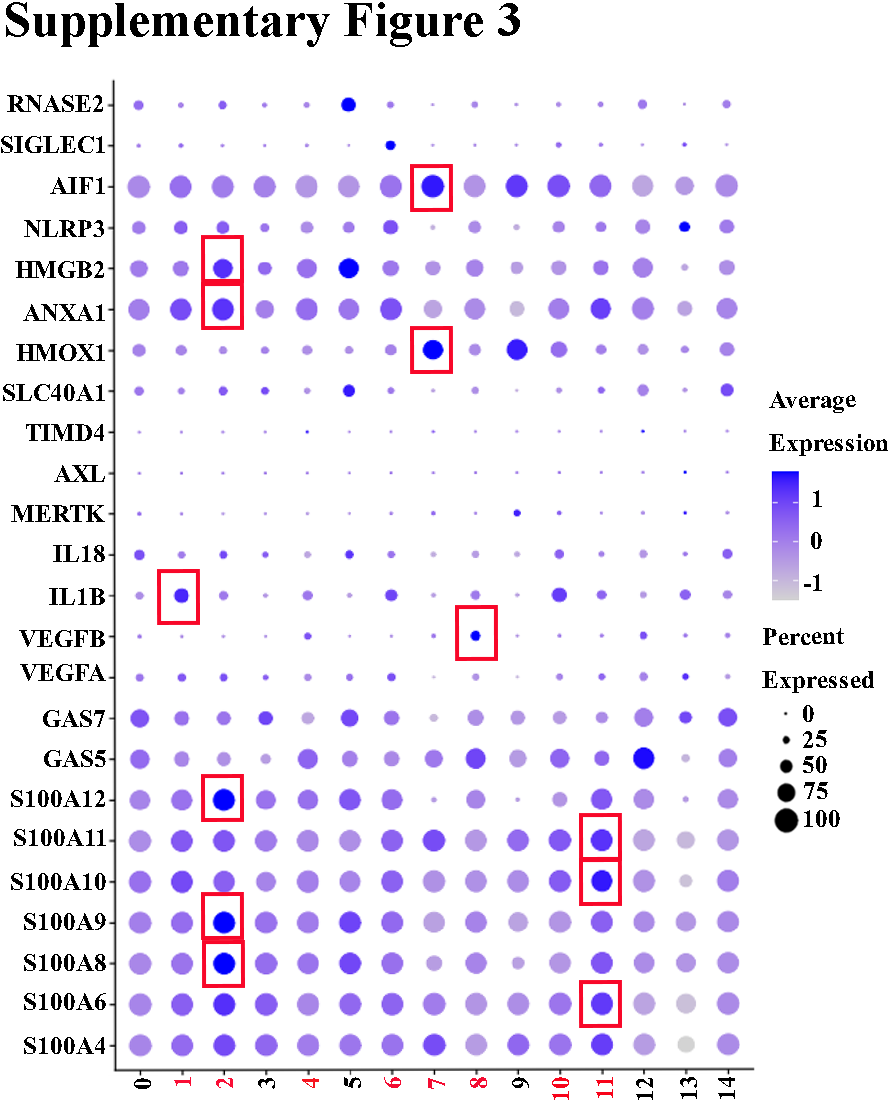

Supplement: Supplementary file 10 — Additional file 10: Figure S3. Dot plots of differentially expressed genes in each cluster. [file 40164_2023_402_MOESM10_ESM.tif]

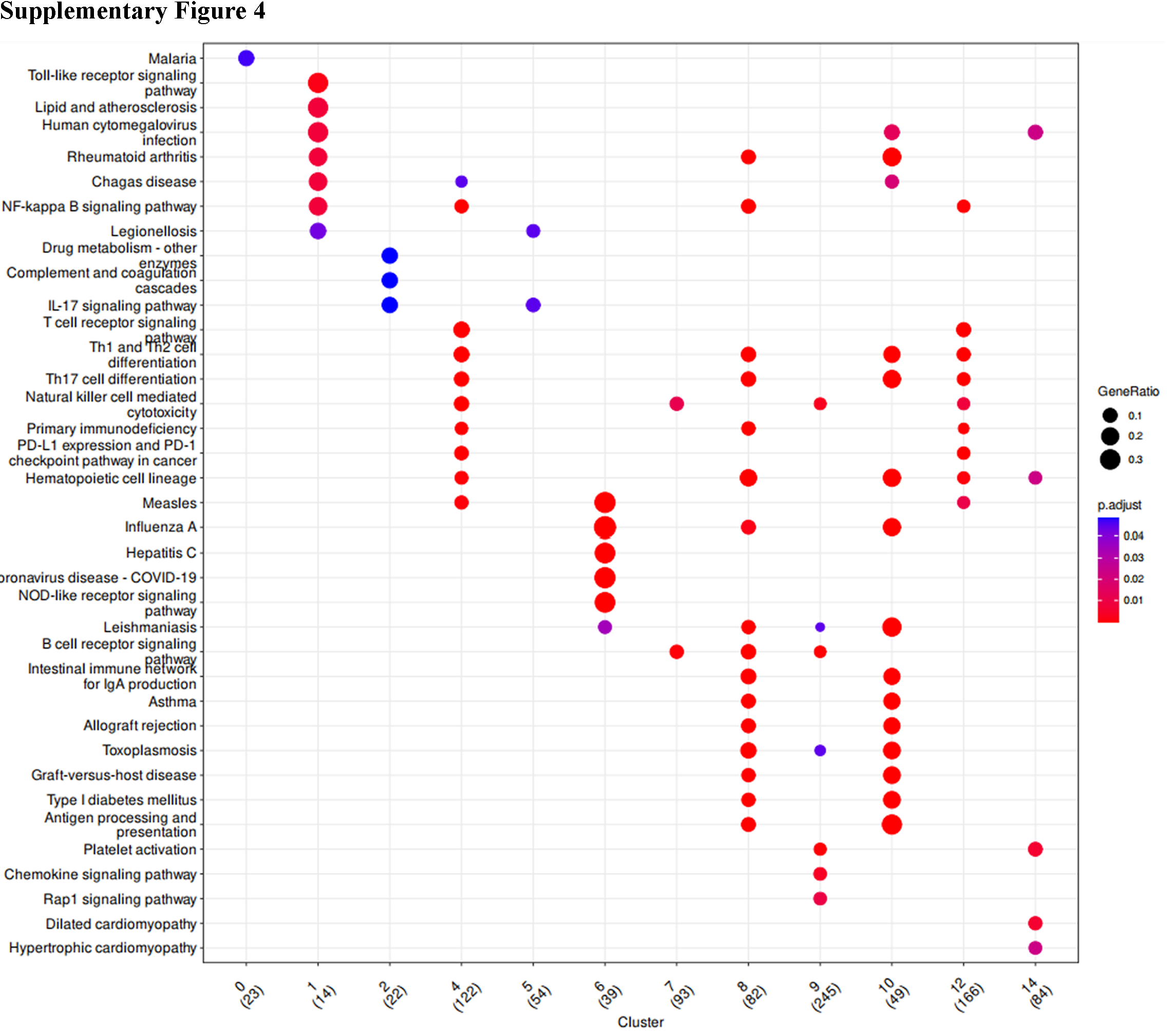

Supplement: Supplementary file 11 — Additional file 11: Figure S4. The enrichment of KEGG pathway in each cluster of monocytes/macrophages. [file 40164_2023_402_MOESM11_ESM.tif]

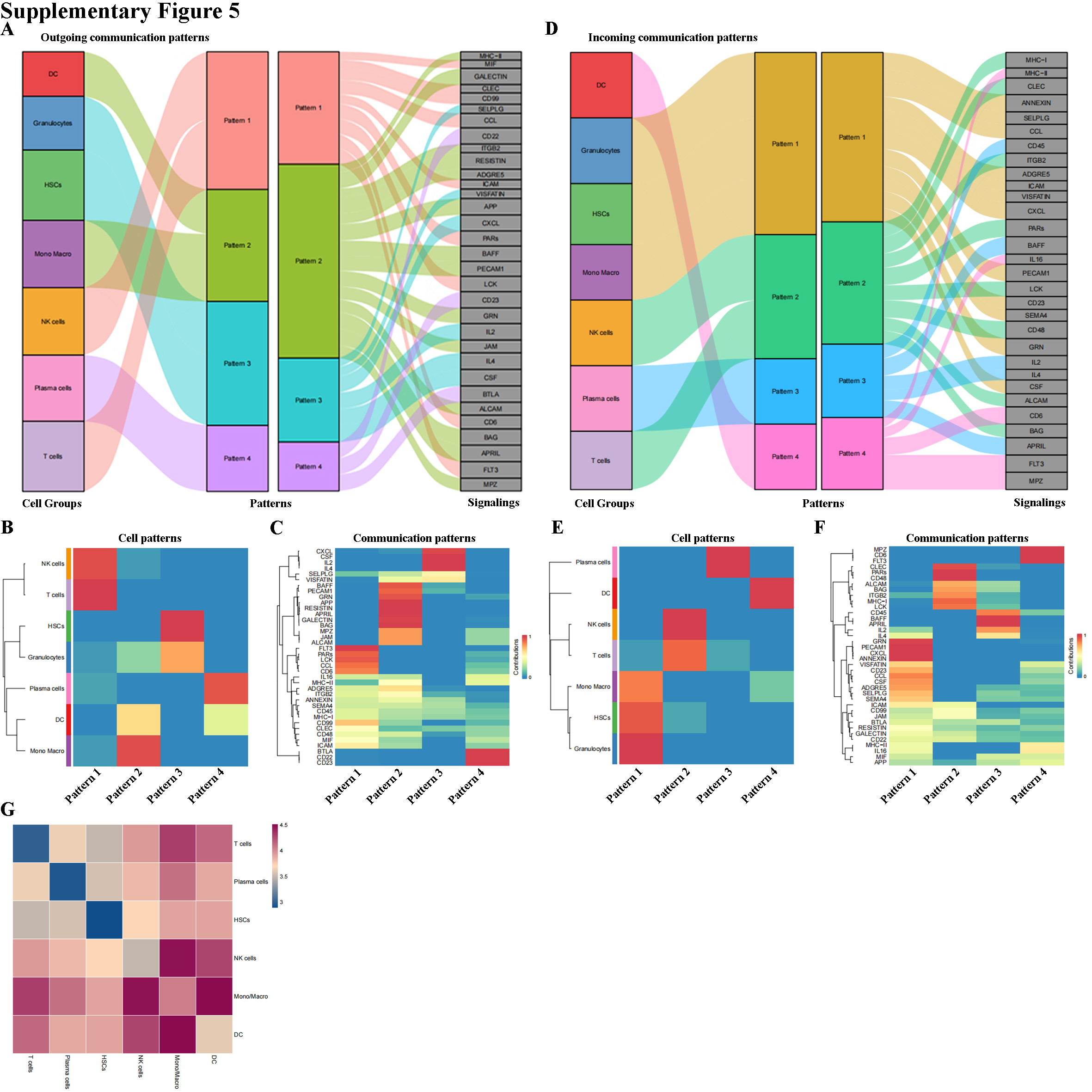

Supplement: Supplementary file 12 — Additional file 12: Figure S5. The communication patterns that connect cell groups with signaling pathways either in the context of outgoing signaling or incoming signaling. [file 40164_2023_402_MOESM12_ESM.tif]
